# Two-Photon Absorption in Twisted Graphene/Hexagonal Boron Nitride Heterojunction Tuned by Vertical Electric Field

**DOI:** 10.3390/nano15050345

**Published:** 2025-02-23

**Authors:** Mengping Chen, Yingliang Chen, Guang Yang, Qiwen Wang, Xiaobo Feng

**Affiliations:** 1 School of Physics and Electronic Information, Yunnan Normal University, Kunming 650500, China; 223090015@ynnu.edu.cn (M.C.); 2333090004@ynnu.edu.cn (Y.C.); 2223090001@ynnu.edu.cn (G.Y.); 2223090005@ynnu.edu.cn (Q.W.); 2 Yunnan Key Laboratory of Opto-Electronic Information Technology, Yunnan Normal University, Kunming 650500, China

**Keywords:** two-photon absorption, graphene/hexagonal boron nitride heterojunction, twist angle, vertical electric field

## Abstract

We theoretically investigate the comprehensive modulation effect of interlayer twisting and external electric field to the two-photon absorption (TPA) in twisted graphene/hexagonal boron nitride (tG/hBN) heterojunction with small twist angles (2° < θ < 10°) starting from an effective continuum model. It is found that the TPA of tG/hBN is extended to the visible light band from infrared light band of that in twisted bilayer graphene (tBLG) due to the increase in energy band gap caused by twisting and the potential energy of the boron nitride atomic layer. And the TPA coefficient is enhanced several times via an external electric field, which increases the density of states, leading to an increase transition probability for two-photon absorption.

## 1. Introduction

Since its preparation in 2004, monolayer graphene has emerged as a research hotspot in condensed-matter physics, owing to its exceptionally high electron mobility, high transparency, and the presence of the room temperature quantum Hall effect [1,2,3,4,5]. However, the zero bandgap of graphene restricts its application in the field of semiconductor optoelectronic devices [6]. As early as 2010, the graphene/hexagonal boron nitride (G/hBN) system was experimentally prepared using boron nitride (BN) as the substrate [7]. Compared to the graphene/silicon dioxide (G/SiO_2_) system, G/hBN not only effectively enhanced the mobility of graphene but also opened the band gap due to the natural mismatch between the graphene and hBN lattices [8]. Owing to its honeycomb-like lattice structure and flat surface, which are akin to those of graphene, hBN stands as one of the optimal substrates for graphene-based nanophotonic devices [9,10,11,12]. The G/hBN heterostructure not only retains some of graphene’s superior electronic properties but also alters its original semi-metallic material characteristics, indicating that G/hBN holds broad application prospects in fields like photodetectors, optical storage, optoelectronic imaging, and so on [13].

In these optoelectronic applications, the optical absorption effect, which is closely related to energy level transitions, is an important channel [14]. Current research on the optical absorption properties of G/hBN indicates that graphene/hBN/graphene heterostructure nanodisk arrays, based on the phonon–plasmon–polariton hybridization theory, can be fabricated into electrically tunable absorbers within and beyond the Reststrahlen band region [15]. Through theoretical simulations, Hodjat Hajian et al. found that G/hBN/G multilayer films exhibit perfect multi-resonance absorption peaks [16]. Among the optical absorption effects, the two-photon absorption (TPA) effect is extensively used because of its advantages in longer excitation wavelength, deeper penetration depth, and higher spatial resolution, compared with one-photon absorption [17].

The interlayer twist of two-dimensional (2D) materials serves as an effective tuning method for optoelectronic devices utilizing 2D material systems, such as G/hBN van der Waals heterojunctions [18]. Twisting can break the degeneracy of AA sites in G/hBN heterojunctions, resulting in additional band gaps, as well as polarization of the conduction and valence bands [19]. By altering the twist angle, the quasi-parabolic band of G/hBN can be hybridized to adjust its bandgap [20]. With the development of modern synthesis technology, tG/hBN Moiré phase with highly convergent interlayer angles has been prepared using a one-pot chemical vapor deposition growth and other methods. The study of tG/hBN heterojunctions has become a reality [21]. In addition to the twist angle, which is a structural parameter of the material itself, an external vertical electric field can also significantly alter the electronic structure of G/hBN heterojunctions, including displacement, cross resistance, and other band deformations [22].

In the present paper, we systematically study the modulation effect of the twist angle and external electric field on the two-photon absorption properties of G/hBN heterojunctions and compared the results with those of bilayer graphene (BLG), revealing the superior performance of G/hBN heterojunctions. We obtain the energy band structure of tG/hBN on the basement of the continuous approximate model in the low-energy theory in the absence/presence of external electric field. It is verified by many studies that the continuous approximate model is an effective way to settle the band structure of the twisted system with relatively small twist angles. Further, we numerically calculate the TPA coefficient and display the TPA spectra as functions of both the twist angle and the external electric field, employing the second-order perturbation theory of electron–photon interaction, which is a commonly used theory for studying two-photon transition processes from a microscopic perspective. The results indicate that the absorption peak of tG/hBN can be extended to the visible light band by twisting, suggesting a potentially broader application prospect for G/hBN in the visible light spectrum compared to BLG. And the TPA coefficient is enhanced several times via an external electric field, which increases the density of states, leading to an increase transition probability for two-photon absorption. These results provide guidance on potential applications of optoelectronic nanodevices based on tG/hBN heterojunction.

## 2. Theoretical Model

**Figure 1:**
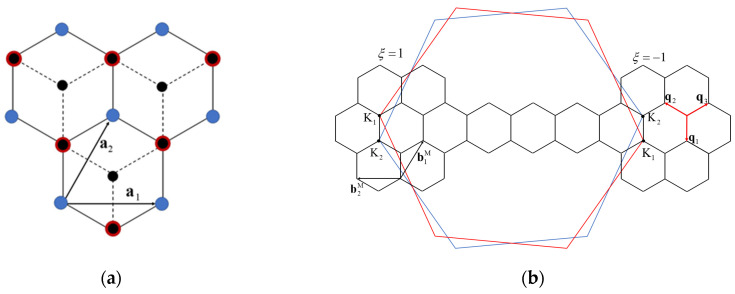
(**a**) Lattice structure of AB-stacking G/hBN heterojunctions, in which boron (nitrogen) atoms are represented by red (blue) circles. Black circles represent carbon atoms in graphene located in the upper layer of hBN layers; (**b**) the Brillouin zone of the twisted G/hBN heterojunction, with blue and red hexagons representing the Brillouin zone of the bottom hBN and top graphene, respectively, and the small hexagons in the middle representing the superlattice Brillouin zone.

hBN is a 2D layer made of nitrogen and boron atoms arranged into a honeycomb structure, and its lattice constant is 0.2504 nm, which is slightly larger than that of graphene’s 0.246 nm, with a lattice mismatch of less than 2% between the two [23,24]. Since the researchers treated the hBN and graphene as two honeycomb structures with the same lattice constant 0.246 nm in many previous theoretical studies on G/hBN, we also adopt this approach in this paper [25]. Figure 1 illustrates the atomic structure of the AB-stacked G/hBN heterojunction and its corresponding Brillouin zone (BZ), as well as the twisted Moiré BZ.

For primitive AB-stacked G/hBN heterojunctions without twisting, the lattice vectors are defined as **a**_1_ = a (1, 0), **a**_2_ = a (1/2, $\sqrt{3}$/2), and the corresponding reciprocal lattice vector **b**_1_ = 2 π/ a (1, –1/$\sqrt{3}$), **b**_2_ = 2 π/a (0, 2/$\sqrt{3}$), with the lattice constant a = 0.246 nm. The first BZ is a hexagon with the two Dirac points at **K**_0*ξ*_ = −*ξ*4 π/3 a (1, 0), where *ξ* = ±1 represents two valley indices. Based on the primitive AB-stacked G/hBN heterojunction, the two layers are twisted with a relative angle *θ* around the boron atoms. When the G/hBN heterojunction is twisted in a specific angle, which is called a commensurate angle, Moiré patterns are formed. For every commensurate twist angle *θ*, it satisfies the following:

(1) $$cos\theta =\frac{3p^{2}+3pq+q^{2}/2}{3p^{2}+3pq+q^{2}},$$

where *p* and *q* are positive integers [26,27]. The primitive lattice vectors and the reciprocal lattice vectors of the Moiré superlattice can be transformed from the untwisted ones with $a_{i}^{M}=R^{-1}a_{i}$ and $b_{i}^{M}=R^{T}b_{i}$ with the rotation matrix

(2) $$R(\theta )=\left(\begin{array}{cc} cos\theta & -sin\theta \\ sin\theta & cos\theta \end{array}\right).$$

The positions of the two Dirac points undergo transformation as a result $K_{\xi }=-(I-R^{T})K_{0\xi }$.

Under the application of a small twist angle, the Moiré superlattice constant is much larger than the atomic scale. So, an effective continuum model similar to the nearly free-electron model in solid-state physics can effectively capture the low-energy electronic structure [25]. In the literature, this effective continuum model has been introduced for twisted bilayer graphene and also for the G/hBN system [23,25]. Under an external vertical electric field, the electronic Hamiltonian in the two-layer basis of graphene and hBN can be expressed as follows [28]:

(3) $$H=\left(\begin{array}{cccc} H_{G} & T_{1}^{†} & T_{2}^{†} & T_{3}^{†}\\ T_{1} & H_{hBN,1} & 0 & 0\\ T_{2} & 0 & H_{hBN,2} & 0\\ T_{3} & 0 & 0 & H_{hBN,3} \end{array}\right),$$

where *H*_G_ and *H*_hBN,*j*_ (*j* = 1, 2, 3) are the Hamiltonians for the isolated graphene and hBN layer described via the Dirac model, which can be expanded in the vicinity of **K**_1*ξ*_ as follows [29]:

(4) $$H_{G}=-\hbar v_{1}k\cdot \sigma_{\xi }+U,$$

(5) $$H_{hBN,j}=-\hbar v_{2}(k+q_{j\xi })\cdot \sigma_{\xi }+mv_{1}^{2}\sigma_{z}-U,$$

where $v_{i}=\sqrt{3}at_{i}/2\hbar$ is the Fermi velocity of each layer with the transition energy between the nearest neighboring atoms in the *i*-th layer *t*_1_ = 2.97 eV, *t*_2_ = 0.39 eV, and *σ_ξ_* = (*ξσ_x_*, *σ_y_*) with the Pauli matrices *σ*. ***k*** represents the wave vectors relative to the Dirac point **K**_1*ξ*_. $q_{j\xi }$ (*j* = 1, 2, 3) connects the Dirac point **K**_1*ξ*_ and three the nearest Dirac points **K**_2*ξ*,_*_j_* around **K**_1*ξ*_ by $q_{1\xi }=K_{1\xi }-K_{2\xi }$, $q_{2\xi }=q_{1\xi }+\xi b_{1}^{M}$, and $q_{3\xi }=q_{1\xi }+\xi (b_{1}^{M}+b_{2}^{M})$.$mv_{1}^{2}\approx 2.3\;eV$ refers to the on-site potentials energy of hBN since the energy band of hBN is gapped and the low-energy spectrum is dominated by graphene [22,25,28]. *U* is the bias energy tuned by the perpendicular electric field *E*. The matrices *T_j_* are the interlayer tunneling, which is formally equivalent to that for twisted bilayer graphene [28]:

(6) $$T_{1}=u_{0}\left(\begin{array}{cc} 1 & 1\\ 1 & 1 \end{array}\right),$$

(7) $$T_{2}=u_{0}\left(\begin{array}{cc} 1 & e^{-i\xi \phi }\\ e^{i\xi \phi } & 1 \end{array}\right)e^{i\xi b_{1}^{M}\cdot r},$$

(8) $$T_{3}=u_{0}\left(\begin{array}{cc} 1 & e^{i\xi \phi }\\ e^{-i\xi \phi } & 1 \end{array}\right)e^{i\xi (b_{1}^{M}+b_{2}^{M})\cdot r},$$

where *ϕ* = 2π/3 and *u*_0_ the interlayer tunneling parameter that reflects the coupling strength between the twisted layers, with a value of 152 meV in the present tight-binding parameters [20]. By diagonalizing the Hamiltonian in Equation (3), the low-energy band structure of the twisted G/hBN system with strain can be obtained. Combining the eigenvalues and wave functions obtained via continuum model, the two-photon generation rate with incident light frequency ω can be represented in the second-order perturbation theory with respect to the electron–photon interaction as follows [30]:

(9) $$W_{2}=\frac{2\pi }{\hbar }\int \sum_{f,i}{\left|M_{f,i}\right|}^{2}\delta (E_{f}-E_{i}-2\hbar \omega )\frac{d^{2}k}{{\left(2\pi \right)}^{2}},$$

(10) $$M_{f,i}=\sum_{m}\frac{\langle \varphi_{f}|H_{int}|\varphi_{m}\rangle \langle \varphi_{m}|H_{int}|\varphi_{i}\rangle }{E_{m}-E_{i}-\hbar \omega },$$

where *φ_i_*_(*f*,*m*)_ and *E_i_*_(*f,m*)_ denotes the initial (final, intermediate) states and energy levels, respectively. *H*_int_ is the electron–photon interaction Hamiltonian, which can be expressed as *H*_int_ = *eħ/*(*m*_e_*c*)**A∙*k*** with electron charge *e*, effective mass me, speed of light in vacuum c, and the light wave vector potential **A** = A**e**. The TPA coefficient *α*_2_, which characterizes the intensity of TPA, is defined by the two-photon transition rate *W*_2_ through the relationship *α*_2_ = 4 *W*_2_*ħω*/ (*I*^2^*d*) with *d =* 3.22 Å the thickness of twisted G/hBN and *I* the intensity of the incident light.

## 3. Results and Discussion

In this study, we focus on investigating the TPA properties of twisted G/hBN system with commensurate twist angles 3.89°, 5°, 6.01°, 7.34°, and 9.43° within the range of 2° < *θ* < 10°, in order to meet the applicable conditions of the continuous effective model [31]. Due to the symmetry of the valleys, we investigate the case where *ξ* = −1 and **K** denotes **K**_−1_ in this research.

**Figure 2:**
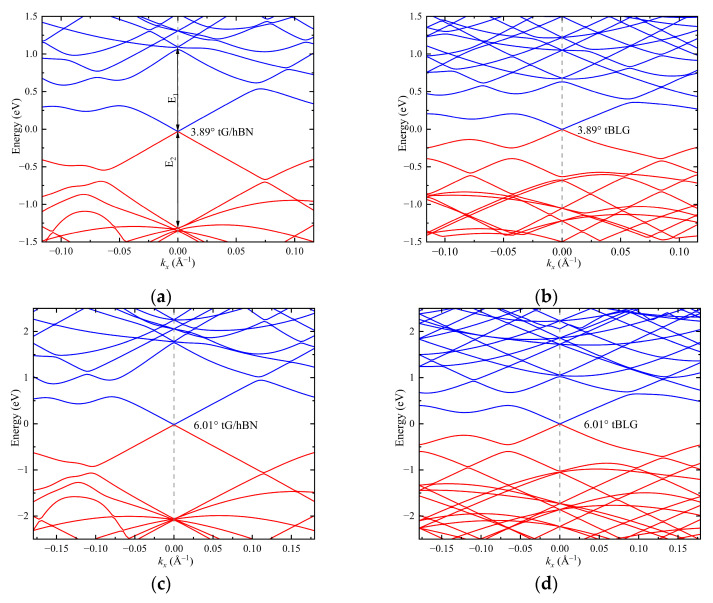

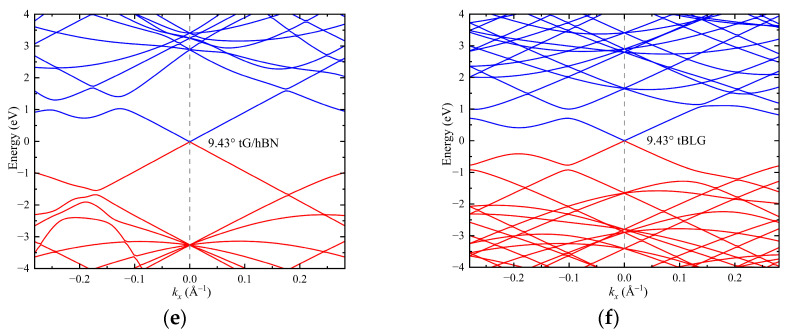
Band structure of twisted G/hBN heterojunction (**a**,**c**,**e**) and tBLG (**b**,**d**,**f**) with different twist angles.

Figure 2 illustrates the energy band structure of twisted G/hBN and tBLG centered around the **K** point along the *k_x_* direction with three different commensurate twist angles, where the vertical dashed line indicates the **K** point. It can be observed that the energy bands of both the two twisted van der Waals materials form Dirac cones at the **K** point. Moreover, as the twist angle increases, the band gap, particularly near the zero energy surface, significantly widens. This implies a reduction in the density of states (DOS). However, unlike tBLG, the sub-bands of the valence band in the twisted G/hBN heterojunction system converge at a point, resulting in a high degree of degeneracy at **K** point, which provide advantages for our subsequent study on the TPA. With the same twist angle, the band gap of twisted G/hBN heterojunction is larger than tBLG.

**Figure 3:**
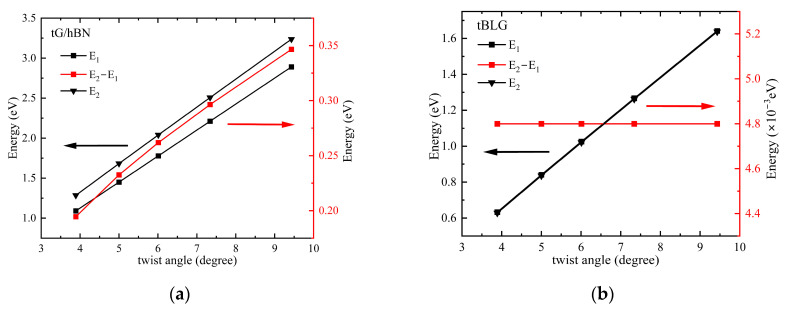
Relationship diagram between energy level difference and twist angle for (**a**) G/hBN and (**b**) BLG.

To more intuitively demonstrate the modulation of band gaps by the twist angle, we plotted the relationship between the two band gaps E_1_ and E_2_ at **K** point (marked in Figure 2) and the twist angle, as illustrated in Figure 3. Since the transition resonance of the TPA necessitates uniformly distributed energy levels, Figure 3 also illustrates the relationship between the twist angle and the band gap difference *E*_2_ − *E*_1_. It is found that the band gap increases linearly with the twist angle. For the twisted G/hBN system, an increase in twist angle results in a larger band gap difference, indicating that the valence band at the **K** point moves away from the zero energy surface faster than the conduction band. In contrast, for tBLG, the band gap difference barely varies with the twist angle. And the conduction and valence bands near the zero energy surface at the **K** point exhibit almost symmetrical distribution. The aforementioned difference arises from the additional potential energy of the underlying hBN atoms in the Hamiltonian of the twisted G/hBN heterojunction [25]. The dissimilarities between the two layers of materials result in varying adjustments to their energy bands with the same twist angle.

**Figure 4:**
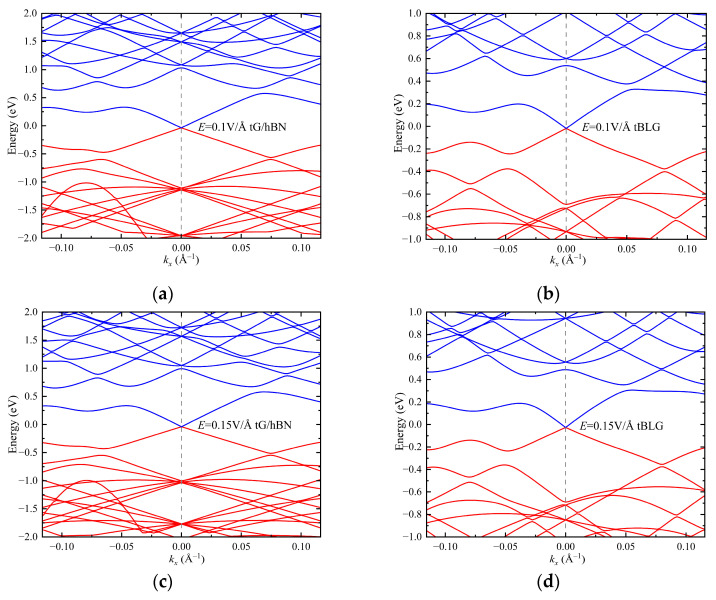

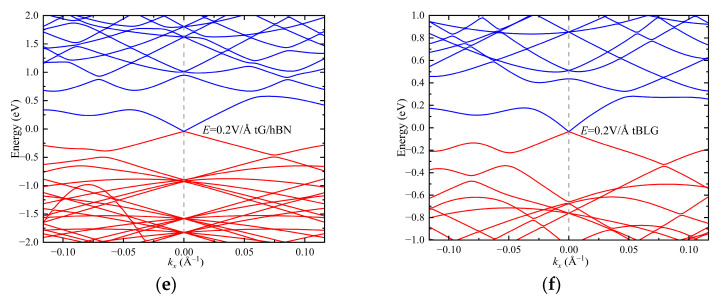
Band structures of twisted G/hBN heterojunction (**a**,**c**,**e**) and tBLG (**b**,**d**,**f**) with twist angle 3.89° under different vertical electric fields.

In order to investigate the influence of the external vertical electric field on the material properties, Figure 4 illustrates the energy band structures of twisted G/hBN heterojunction and bilayer graphene with a twisting angle of 3.89° under electric field intensities of 0.1 V/Å, 0.15 V/Å, and 0.2 V/Å. As the applied electric field intensity increases, for the twisted G/hBN heterojunction, the modulation effect on the conduction band energy levels is not significant. However, the valence band energy levels gradually shift towards higher energies, resulting in band convergence at several points near the **K** point. For tBLG, the vertical electric field exerts minimal influence on it. This phenomenon is precisely the combined result of the screening effect and field-induced band structure modification [32,33]. Under the application of external electric field, the screening effect causes the electrons redistribution to counteract the field. But the field-induced band structure modification partially suppresses the screening effect by altering the density of states simultaneously.

**Figure 5:**
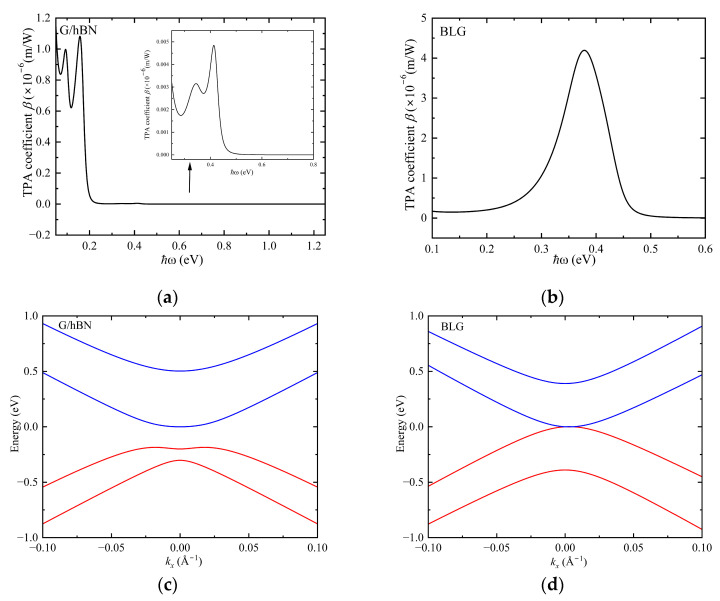
Two-photon absorption spectra and band structures of G/hBN (**a**,**c**) and BLG (**b**,**d**) around K point without twisting and external electric field.

Based on the Hamiltonian of the G/hBN heterojunction and BLG in the absence of twisting and external electric field [22,30], Figure 5 illustrates their energy band structures and TPA spectra. For BLG, a strong absorption peak emerges in the near-infrared band (around 3100 nm) with a magnitude of 10^−6^ m/W, aligning with the findings in Ref. [30], albeit with a slightly lower order of magnitude. This discrepancy arises from the fact that some transition parameters in the Hamiltonian were not overlooked during our calculation. G/hBN exhibits two notable absorption peaks in the far-infrared band (around 13,777 nm and 8266 nm), matching the order of magnitude observed in graphene. The TPA transition predominantly takes place between the valence band and the conduction band proximate to the Fermi surface. Notably, G/hBN’s energy band spacing is narrower than that of bilayer graphene, consequently leading to its absorption peak occurring at a lower energy range (specifically, in the far-infrared region) compared to BLG. Moreover, a minor absorption peak is observed at 0.4 eV (near 3100 nm), attributed to the underlying graphene layer, which preserves certain graphene characteristics in G/hBN. Overall, G/hBN demonstrates superior TPA capabilities in mid-infrared and far-infrared regions compared to BLG. This underscores G/hBN’s suitability as a material for two-photon microscopy [34].

**Figure 6:**
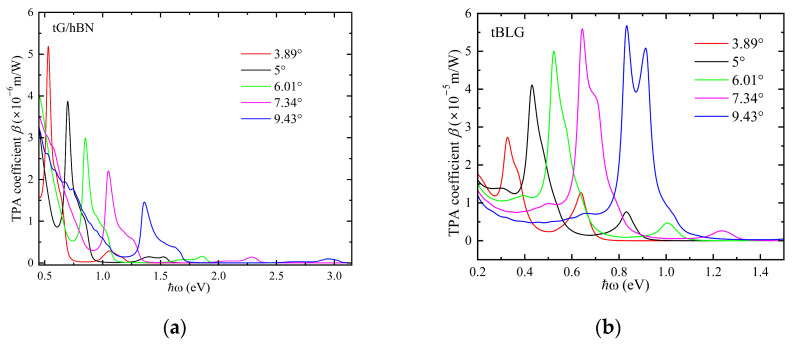
Two-photon absorption spectra of tG/hBN (**a**) and tBLG (**b**) with different twist angles around **K** point.

Figure 6 illustrates the TPA spectra of G/hBN and BLG across several twist angles. As the twist angle increases, both G/hBN and BLG exhibit a blue shift in their TPA peaks, attributed to the widening of the band gap spacing due to the increasing twist angle. Notably, the peak value of the TPA peak for G/hBN diminishes with increasing twist angle, whereas the peak value for BLG increases. This disparity arises from the fact that, as discussed above, G/hBN’s valence band is more responsive to the twist angle, leading to an imbalance between the conduction and valence band gaps and reducing the likelihood of two-photon resonant transitions. This imbalance stems from the potential energy difference between the two atomic layers. Conversely, for BLG, the twist angle exerts minimal influence on the regulation of its conduction and valence bands, preserving balanced energy band spacing. At equivalent twist angles, G/hBN’s absorption peak is positioned at higher energies compared to BLG’s. Remarkably, at significant twist angles, G/hBN’s absorption peak even falls within the visible light range (1.65 eV to 3.1 eV), suggesting a potentially broader application prospect for G/hBN in the visible-light spectrum compared to BLG at large twist angles.

**Figure 7:**
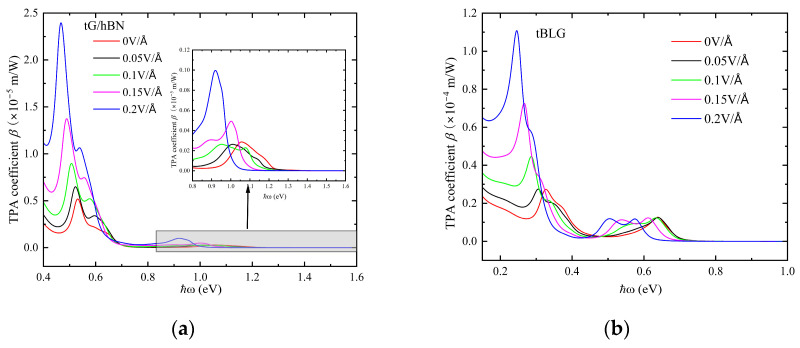
Two-photon absorption spectra of tG/hBN (**a**) and tBLG (**b**) with twist angle 3.89° under different vertical electric fields.

We selected a twist angle of 3.89° to further investigate the impact of applied electric field strength on the TPA effect in twisted G/hBN heterojunctions and tBLG, as illustrated in Figure 7. It can be observed that, under an external electric field, the TPA coefficients of both material systems are enhanced compared to those in the absence of the external electric field. Furthermore, the stronger the electric field intensity, the greater the TPA coefficient. Specifically, the peak value of the TPA coefficient at electric field strength of 0.2 V/Å is nearly five times greater than that in the absence of external field. This is attributed to the field-induced band structure modification, which reduces the energy band gap and increases the density of states, leading to an increase in the number of electronic energy states for two-photon transitions. Moreover, the position of the absorption peak shifts to the red with the increase in the electric field strength. Similar results were obtained at other commensurate twist angles. We collected some experimental data on two-photon absorption in other similar systems, which are listed in Table 1. The deviation from the experimental data is primarily due to the influence of sample concentration on the experimental results. The discrepancy with the theoretical calculation data from Ref. [31] is mainly because it employed a four-band model when calculating the band structure.

## 4. Conclusions

In this paper, we depict the TPA properties of tG/hBN with and without an electric field and compare it with that of tBLG based on the effective continuum model with the twisted angle in the range of 2° < *θ* < 10° and the second-order perturbation theory of electron–photon interaction. It is found that the two-photon optical absorption of G/hBN without twisting occurs in the far-infrared and mid-infrared light band, which is much wider than that of BLG. As the twist angle is increased, the TPA peaks of tG/hBN are blue-shifted and even to the visible light band, which further proves that the optical absorption range of tG/hBN is wider than that of tBLG. The external electric field can enhance the TPA effect by several times, and the stronger the electric field intensity, the greater the TPA coefficient due to the higher density of states and transition probability for two-photon absorption. In future works, we can validate our findings combined with the Z-Scan technique.

## Figures and Tables

**Table 1:** TPA coefficients of similar systems using this theory and other theories, as well as experimental data for comparison.

Sample	Electric Field	Twist Angle (Degree)	Wavelength (nm)	β (Experiment) (m/W)	β (Other Theory) (m/W)	β (This Theory) (m/W)
BLG	0	0	3100	2 × 10^−3^ [35]	2 × 10^−3^ [35]	
hBN/GO nanosheets	0	0	532	(1.34 ± 0.20) × 10^−8^ [36]		
hBN nanosheets	0	0	532	(1.14 ± 0.70) × 10^−8^ [36]		
BLG	0	0	3100		3.8 × 10^−4^ [31]	
tBLG	0	3.89	3100		3 × 10^−6^ [31]	
tBLG	0	7.34	3100		3.2 × 10^−6^ [31]	
tBLG	0	13.17	3100		3.2 × 10^−6^ [31]	
TLG	0	0	2200		1.2 × 10^−4^ [31]	
tTLG	0	3.89	3100		2.5 × 10^−4^ [31]	
tTLG	0	7.34	3100		2.4 × 10^−4^ [31]	
tTLG	0	13.17	2200		2 × 10^−4^ [31]	
tDBLG	0	3.89	3100		3.6 × 10^−4^ [31]	
tDBLG	0	7.34	3100		3.5 × 10^−4^ [31]	
tDBLG	0	13.17	3100		3 × 10^−4^ [31]	
BLG	0	0	3100			4.1 × 10^−6^
tBLG	0	3.89	3870			2.8 × 10^−5^
tBLG	0	7.34	1650			5.5 × 10^−5^
tBLG	0.1 V/Å	3.89	4100			4.1 × 10^−5^
G/hBN	0	0	6200			1.1 × 10^−6^
tG/hBN	0	3.89	2480			5.1 × 10^−6^
tG/hBN	0	7.34	1033			2.2 × 10^−6^
tG/hBN	0.1 V/Å	3.89	2480			9 × 10^−6^

## Data Availability

The original contributions presented in this study are included in the article. Further inquiries can be directed to the corresponding author.

## Abbreviations

G/hBN	graphene/hexagonal boron nitride
hBN	hexagonal boron nitride
tG/hBN	twisted graphene/hexagonal boron nitride
BLG	bilayer graphene
BZ	Brillouin zone
tBLG	twist bilayer graphene
TPA	two-photon absorption
2D	two-dimensional
G/SiO_2_	graphene/silicon dioxide
GO	graphene oxide
TLG	trilayer graphene
tTLG	twisted trilayer graphene
tDBLG	twisted double-bilayer graphene
